# Joint transcriptomic and metabolomic analyses of the mechanisms of *Juniperus tibetica* in response to *Arceuthobium oxycedri*

**DOI:** 10.1093/aobpla/plag022

**Published:** 2026-05-21

**Authors:** Hongping Chen, Luchao Bai, Yingtai Cao

**Affiliations:** College of Agriculture and Animal Husbandry, Qinghai University, 251 Ningda Road, Chengbei District, Xining City, Qinghai Province 810016, China; College of Agriculture and Animal Husbandry, Qinghai University, 251 Ningda Road, Chengbei District, Xining City, Qinghai Province 810016, China; Industrial Development Division, Huzhu County Agriculture, Rural Affairs, and Science & Technology Bureau, 27 South Street, Weiyuan Town, Huzhu County, Haidong City, Qinghai Province 810500, China

**Keywords:** transcriptome, metabolome, physiological response

## Abstract

As a key species in China's ecologically fragile Sanjiangyuan region, *Juniperus tibetica* faces severe threats from *Arceuthobium oxycedri*. This study preliminarily identified the mistletoe species parasitizing *J. tibetica* as *A. oxycedri*. The results of physiological parameter measurements indicate that infection with *A. oxycedri* significantly increased the activity of peroxidase (POD), catalase (CAT), polyphenol oxidase (PPO), phenylalanine ammonia-lyase (PAL), cinnamaldehyde dehydrogenase (CAD), glutathione peroxidase (GSH-Px), as well as the malondialdehyde (MDA) and proline (PRO) content in *J. tibetica*. This suggests that, under *A. oxycedri* infection, *J. tibetica* resists the infection by scavenging reactive oxygen species, increasing lignin content and enhancing drought-related physiological indicators. Multi-omics analysis indicates that following infection by *A. oxycedri*, the major enriched pathways in *J. tibetica* associated with the *A. oxycedri* invasion are all linked to drought stress defence. It is hypothesized that the growth and reproduction of *A. oxycedri* require the uptake of large amounts of water from the host, its parasitism leads to an imbalance in the host’s water transport, thereby triggering drought-stress-related response mechanisms. These findings are consistent with the results of physiological parameter measurements. This study provides preliminary insights into the physiological and molecular mechanisms by which *J. tibetica* responds to *A. oxycedri* stress, offering a theoretical basis for the effective control of the occurrence and damage caused by *A. oxycedri*.

## Teaser

This study explores the physiological and molecular mechanisms of Juniper responding to dwarf mistletoe infection. The infection disrupts the host’s water transport balance and triggers a series of physiological responses closely associated with drought stress, revealing the core adaptive regulation strategy of host plants under parasitic stress.

## Introduction

*Juniperus tibetica* is a deciduous tree belonging to the genus Juniperus L. within the family Cupressaceae. It is one of the main forest species in the alpine habitat of Qinghai Province because of its cold, drought, and barrenness resistance, and is also an endemic Juniperus L. plant in China. Its community plays an important role in water conservation, species preservation and maintenance of forest ecosystem stability (Ning et al. 2021). *Juniperus tibetica* is infested by *Arceuthobium oxycedri*, which will produce ‘broom clumps’, resulting in reduced growth and even death of the host. *Arceuthobium oxycedri* has the widest natural distribution of all dwarf mistletoe parasites, spanning the Mediterranean region of Europe and northern Africa, near the eastern Himalayas and in western China (Tibet) from central Spain eastwards (Hawksworth et al. 1976). *Arceuthobium oxycedri* can infest several juniper genera in its natural range, as well as other cypress family plants that have been introduced into its natural range (Hawksworth 1996). In China, cypress parasites are mainly found in mountain round cypress forests in Tibet and northwestern Yunnan at altitudes of 3000–3500m (Qiu and Lin 1988). At present, the control of *A. oxycedri* mainly includes agricultural measures, chemical control and biological control (Knutson et al. 1972, Scherm and Yang 1996, Zhou et al. 2007, Li et al. 2010, Xia et al. 2010, Hu et al. 2014, Zhao and Tian 2024), but the control effect is unsatisfactory and difficult to eradicate. Therefore, it is important to explore the molecular mechanism of *J. tibetica* in response to *A. oxycedri*, and to excavate the defence-related genes, so as to lay a certain theoretical foundation for the prevention and control of *A. oxycedri* in the later stage of the research.

In recent years, RNA-seq technology has been widely used in the study of host plant-parasite plant interaction mechanism, for example, signal transduction, programmed cell death, lignin biosynthesis, secondary cell wall formation, and phenylpropane biosynthesis were usually found to be associated with host plant resistance to host plant infestation in the study (Huang et al. 2012, Bai 2020, Yang et al. 2020, Zhang 2023). Wahid et al. had used suppression subtractive hybridization (SSH) technology to damaged and unaffected dwarf mistletoe stem segments to construct forward (F-SSH) and reverse (R-SSH) libraries, respectively, and performed deep sequencing and bioinformatics analyses of the potential roles of dwarf mistletoe responsive genes in a variety of important functions related to bioregulation, metabolic processes, defences, signalling pathways, growth and development, transcription factors, and transporter activity in plants (Wahid et al. 2019). Quantitative analysis of all metabolites produced by plants, i.e. plant metabolomics technology, yields metabolite results that provide a more intuitive picture of plant phenotypic traits. In order to comprehensively investigate the mechanisms related to the response of *J. tibetica* to *A. oxycedri*, differentially expressed genes and differential metabolites can be analysed by transcriptome sequencing in conjunction with metabolomics techniques, which can help to accurately reveal the regulatory network of metabolic pathways in the plant (Xue et al. 2020). Research on physiological indicators of dwarf mistletoe showed that dwarf mistletoe parasitism significantly reduced the length of spruce new shoots, the length of needles, and the hundred-leaf weight of leaves, and the number of new lateral shoots of spruce significantly increased, and the growth of spruce new shoots requires a large amount of water, and the spruce is subjected to spruce dwarf mistletoe infestation, which leads directly to the increase of internal malondialdehyde content (Lu 2020). Currently, fewer studies have been reported on the accumulation of key genes and key metabolites based on the combined transcriptomic and metabolomic analysis of the response of Juniper to dwarf mistletoe. This study analysed gene expression and metabolites in infected and healthy branches by measuring relevant physiological indicators and employing a combined transcriptomic and metabolomic analysis approach. The study aims to elucidate the gene expression patterns and metabolic changes associated with the defence mechanisms of Juniper against dwarf mistletoe, thereby providing a theoretical basis for a deeper understanding of the interactions between Juniper and dwarf mistletoe, and laying the theoretical groundwork for future research into the control of dwarf mistletoe in Juniper.

## Results

### Morphological and molecular identification of Dwarf Mistletoe

Dwarf mistletoe was found on *J. tibetica* in Baizha Forest, Nangqian County, Yushu Tibetan Autonomous Prefecture, Qinghai Province, with flowering and seed ejection occurring from September to November each year. The disease infested *J. tibetica* branches and trunks, occurring mainly on the lateral branches of *J. tibetica*, and no dwarf mistletoe branches were seen in the main trunks and host vigour.

**Figure 1:**
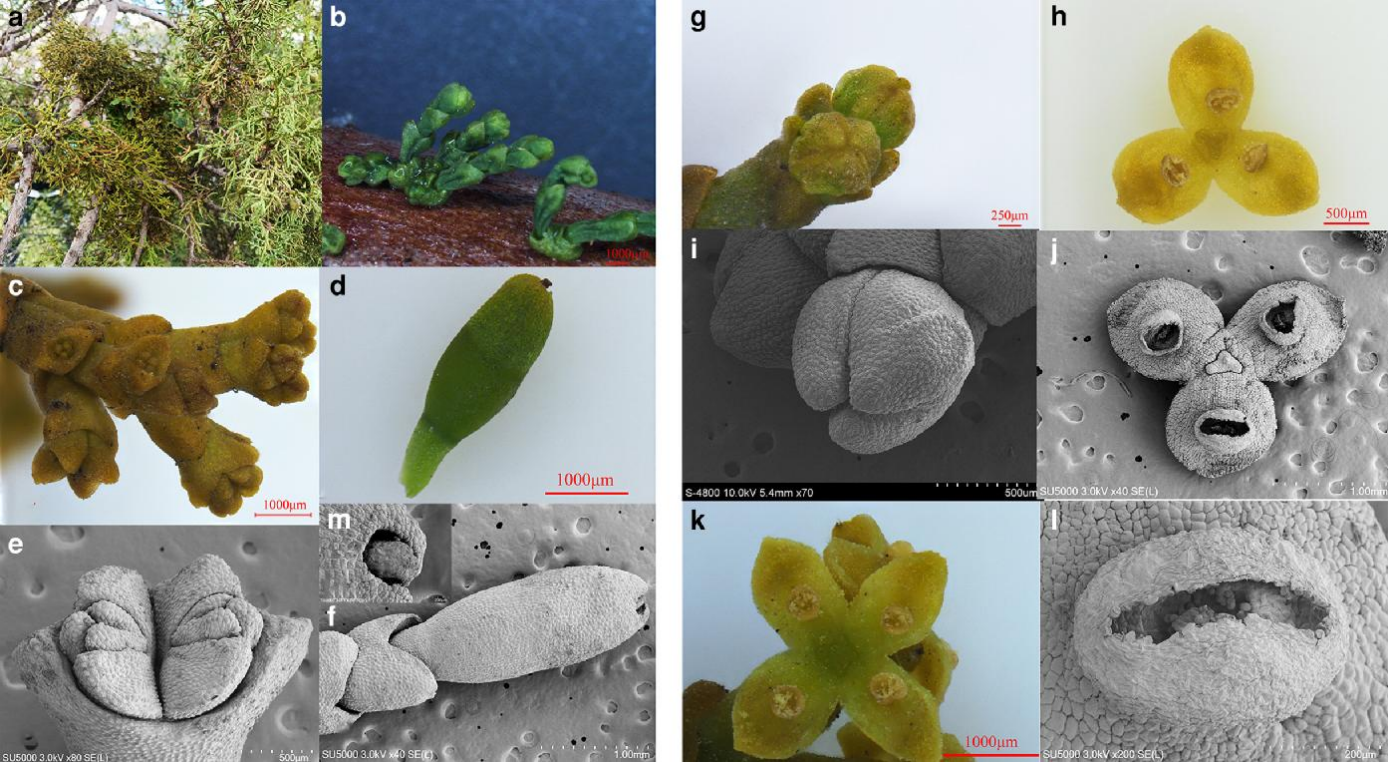
Morphology of dwarf mistletoe. (a) Whole plant; (b) partial (SM); (c) stems (SM); (d) fruits (SM); (e) stems (SEM); (f) fruits (SEM); (g) flower buds (SM); (h) staminate flowers (SM); (i) flower buds (SEM); (j) staminate flowers (SEM); (k) pistillate flowers (SM); (l) anthers (SEM); (m) persistent calyx (partial magnification).

Dwarf mistletoe shoots 2–25 cm high, branches yellowish-green (Fig. 1b); internodes of main stem 5–10 mm long, 1.5–5 mm thick; branches commonly whorled (Fig. 1c and e). Flowers often single or 2–3 on top of short lateral branches, yellowish-green, ovoid in bud, 1–1.5 mm long (Fig. 1g and i), 2–2.5 mm in diam. at anthesis, sepals 3 (Fig. 1h and j), sometimes 4, oblong-ovate, 1–1.4 mm long (Fig. 1k); anthers orbicular, ca. 0.5 mm diam. ca. 1.5 mm in diameter, the upper half surrounded by the persistent calyx (Fig. 1m), the lower half smooth (Fig. 1d); fruiting pedicel ca. 1 mm long (Figure 1f).

The sequences were amplified using primer pairs for ITS, YCf2 and matk genes, and the sequencing results showed that the full length of the ITS sequence of dwarf mistletoe was 185 bp, the full length of the YCf2 sequence of dwarf mistletoe was 174 bp, and the full length of the YCf2 sequence of dwarf mistletoe was 143 bp. A comparison of the ITS sequence (PZ072304) with sequences in GenBank revealed a similarity of 98.86% with A. oxycedri (GenBank accession number HQ917109). A comparison of the YCf2 sequence (PZ121765) with sequences in GenBank it showed a similarity of 96.89% with A. oxycedri (GenBank accession number MN011751). When the matk sequence (PZ121764) was compared with sequences in GenBank, it showed a similarity of 96.18% with A. oxycedri (GenBank accession number MH390653).

Through a combination of morphological and host plant analyses, supplemented by molecular biological studies, the species *A. oxycedri*, which parasitizes *J. tibetica*, has been identified. By comparing the research findings with the description of *A. oxycedri* in the Flora of China, and based on the taxonomic approach for the *Arceuthobium* genus that uses host plants as the basis for classification (as outlined in the Key to Subspecies of the *Arceuthobium* genus in China), it has been preliminarily determined that the parasite found on *J. tibetica* is *A. oxycedri*.

### Physiological indicator response

**Figure 2:**
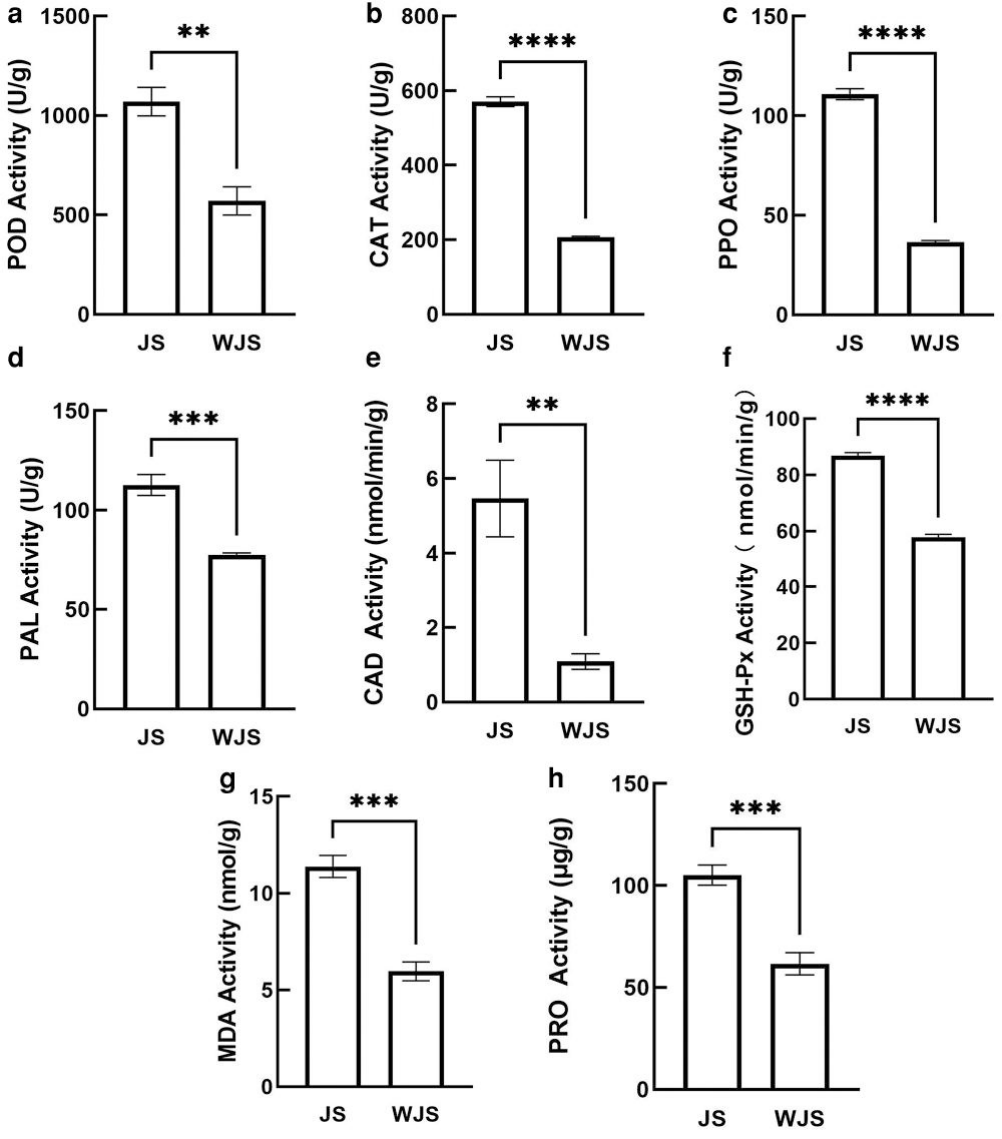
Physiological changes of Sabina chinensis materials infested with *Arceuthobium oxycedri*. *, **, ***, and **** represent JS compared with WJS, respectively The significance levels are *P* < .05, *P* < .01, *P* < .001 and *P* < .0001, respectively.

Compared with the controls, the physiological indicators of branches and stems from infected materials were all higher than those from corresponding uninfected juniper branches and stems to varying degrees (Fig. 2). Among these, the POD and CAD activities in the JS group showed significant differences at *P* < .01 (Fig. 2a and e), while CAT, PPO, and GSH-Px activities exhibited significance at *P* < .0001 (Fig. 2b, c and f). PAL activity and MDA, PRO content showed significance at *P* < .001 (Fig. 2d, g and h).

### Quality assessment and quality control analysis of transcriptomic data

A total of six samples from three replicates each of infested (JS) and healthy (WJS) branches were sequenced for transcriptome libraries, and a total of 140 219 291 Clean reads were obtained in the six samples, with a range of 22.4 million to 24.74 million Clean reads per sample, and the total number of bases in the Clean Data was 42 017 571 812. The range of Q20 was 99.32–99.53%, the range of Q30 was 97.21–97.96%, and the range of GC content was 43.38–43.87% (Table 1), which indicated that the transcriptome sequencing was of high quality, and could be used for subsequent transcriptome splicing and assembly. The sample correlation heatmap and principal component analysis results indicate that the samples exhibit good biological reproducibility (see Supplementary Appendix A for details), and the sequencing data meet the requirements for subsequent bioinformatics analysis.

**Table 1:** Quality control for transcriptome sequencing.

Sample	Clean reads	Clean bases	Q20(%)	Q30 (%)	GC (%)
JS-a1	23 579 973	7 065 544 914	99.43	97.77	43.77
JS-a2	22 915 539	6 866 311 690	99.5	97.94	43.68
JS-a3	23 017 955	6 897 453 304	99.43	97.74	43.87
WJS-b1	23 561 369	7 060 813 556	99.53	97.96	43.4
WJS-b2	22 400 647	6 712 453 720	99.47	97.88	43.45
WJS-b3	24 743 808	7 414 994 628	99.32	97.21	43.38

### Analysis of differentially expressed genes

**Figure 3:**
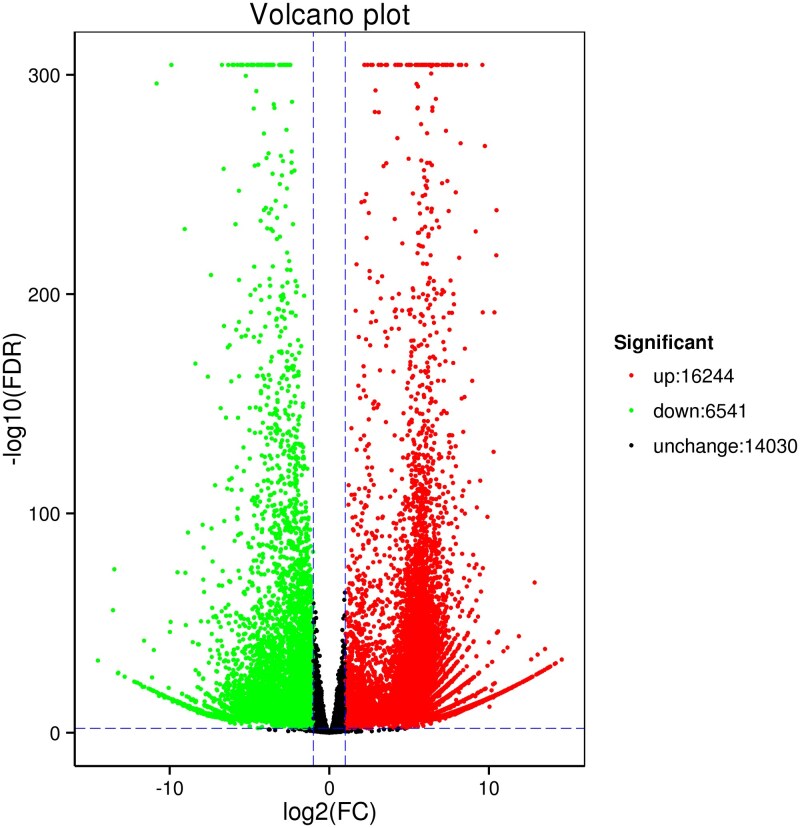
Differentially expressed genes.

A total of 22 785 differentially expressed genes (DEGs) were screened for differential gene expression between the control group (WJS) and the test group (JS) by DESeq software, of which 16 244 differentially expressed genes were up-regulated, accounting for 71.3%, and 6541 differentially expressed genes were down-regulated, accounting for 28.7% (Fig. 3).

## Differential gene Gene Ontology enrichment analysis

**Figure 4:**
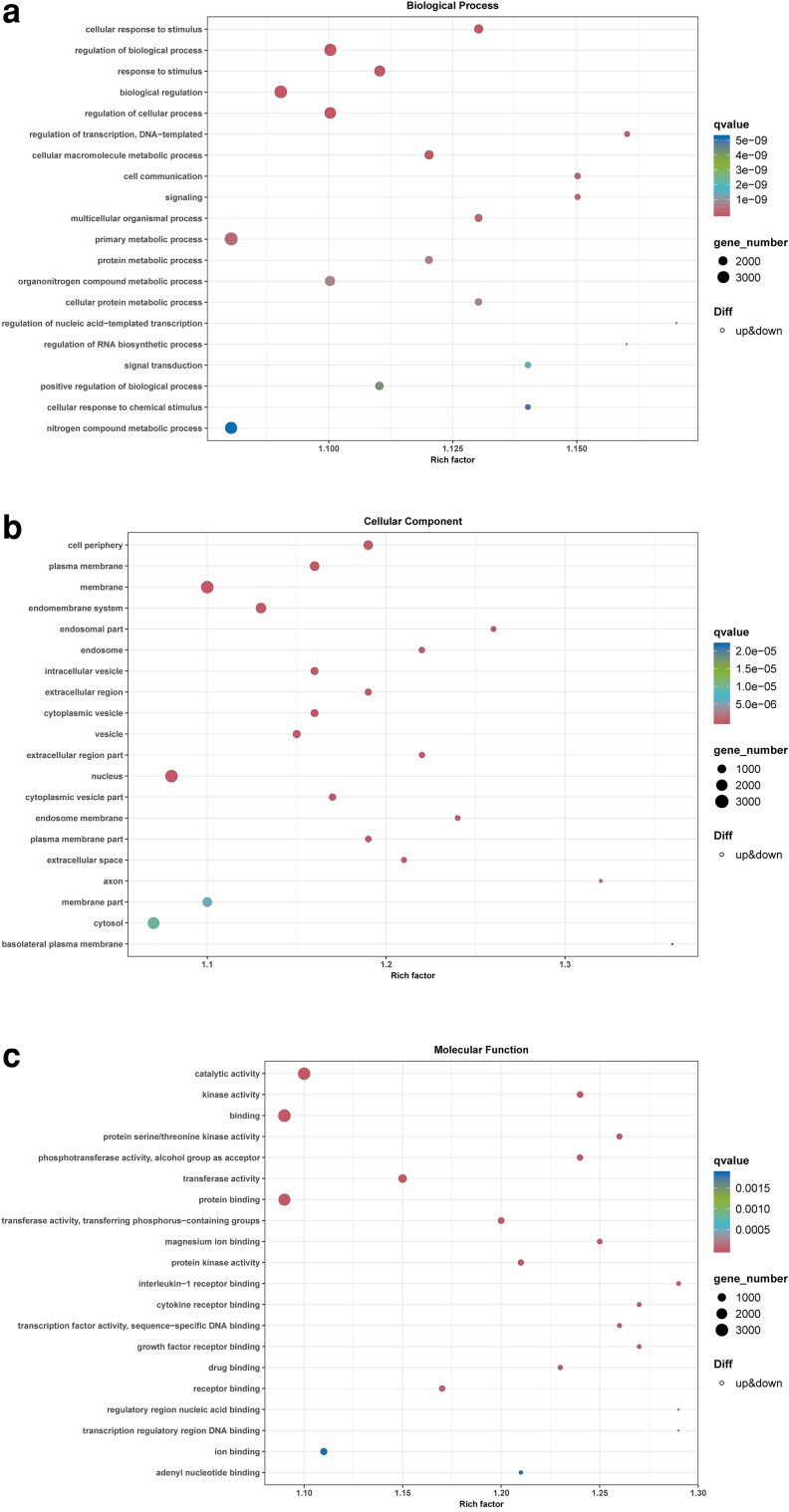
GO enriched analysis on DEGs. (a) biological process; (b) cellular components; (c) molecular functions.

Analysis of the 13 530 DEGs annotated to the corresponding GO secondary nodes revealed that GO entries were enriched to Biological Process, Cellular Component, and Molecular Function (Fig. 4). Enrichment analyses were performed for Biological Process, Cellular Component, and Molecular Function, respectively, and the top 20 enriched GO enrichment results were plotted as bubble plots. The DEGs in biological processes are mainly involved in cellular response to stimulus, regulation of biological process, response to stimulus, biological regulation, regulation of cellular process, regulation of transcription, DNA-templated, cellular macromolecule metabolic process, cell communication, signalling, multicellular organismal process and primary metabolic process. The DEGs in cellular components are mainly involved in cell communication, signalling, multicellular organismal process and primary metabolic process (Fig. 4a). The DEGs are mainly involved in cell periphery, plasma membrane, membrane, endomembrane system, endosome and intracellular vesicle and other cellular components (Fig. 4b). The DEGs in molecular function are mainly involved in catalytic activity, acting on a protein, catalytic activity, kinase activity, binding, protein serine/threonine kinase activity, phosphotransferase activity, alcohol group as acceptor, transferase activity, protein binding, transferase activity, transferring phosphorus-containing groups, and magnesium ion binding (Fig. 4c).

GO enrichment analyses showed that *J. tibetica* was infested with a high number of genes involved in the regulation of biological processes and cellular response to stimuli.

### Differential gene Kyoto Encyclopedia of Genes and Genomes enrichment analysis

**Figure 5:**
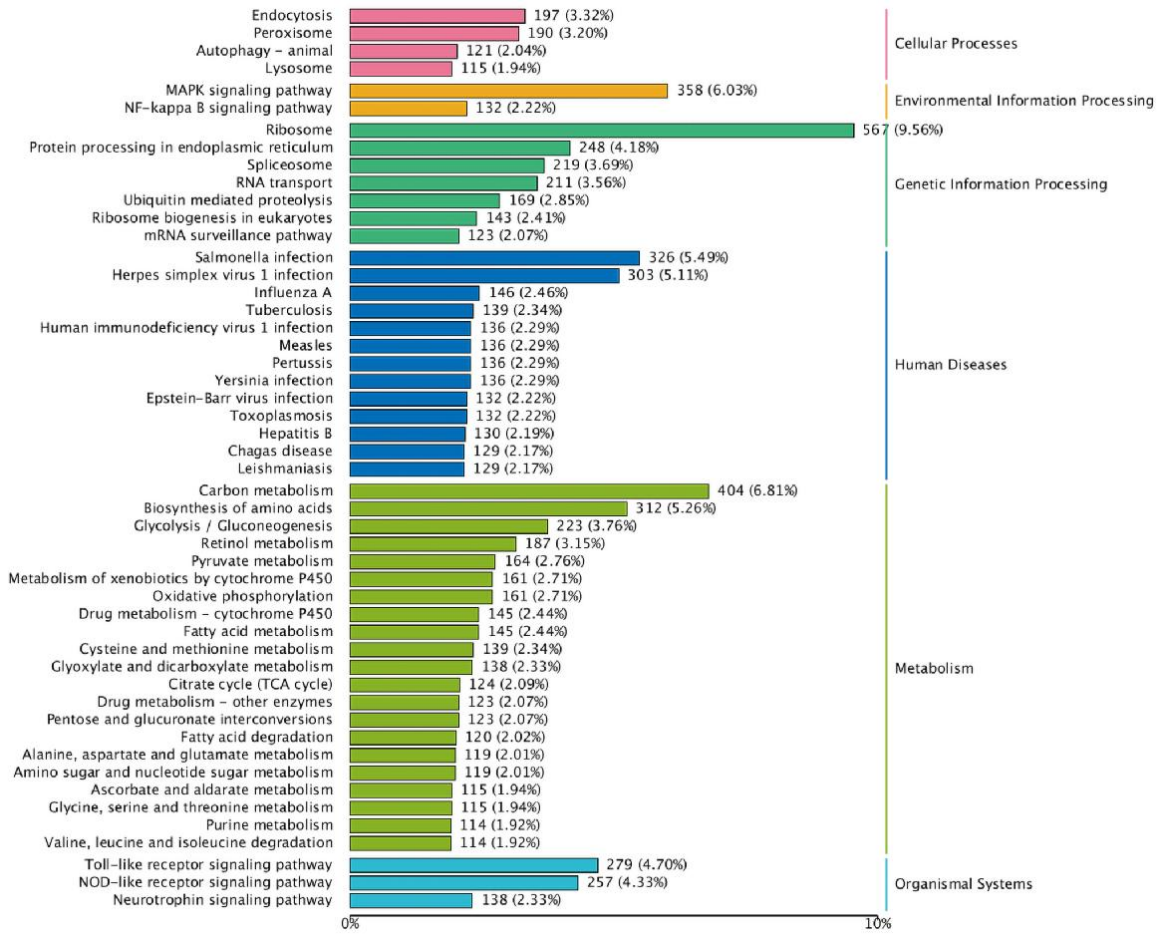
KEGG annotation of differentially expressed genes.

Among the types of biochemical reactions involved in KEGG metabolic pathway, the metabolism type accounts for the largest proportion, of which the top three pathways are carbon metabolism, biosynthesis of amino acids and glycolysis/gluconeogenesis; Additionally, in the type of genetic information processing accounts for a larger proportion, which is dominated by ribosome, protein processing in endoplasmic reticulum, and environmental information processing. In the type of environmental information processing, the MAPK signalling pathway is the main one; in the type of cellular processes, endocytosis is the main one. According to the results of KEGG enrichment analysis, 15 319 DEGs out of 22 785 DEGSs were enriched in 324 metabolic pathways, and the more highly enriched pathways included ribosome, carbon metabolism, and MAPK signalling pathway, biosynthesis of amino acids and protein processing in endoplasmic reticulum, and were enriched to 567, 404,358,312, and 248 DEGs, respectively (Fig. 5). The enrichment results indicated that dwarf mistletoe mainly caused complex molecular biological effects on the MAPK signal transduction pathway of *J. tibetica*, which might be a stress response of *J. tibetica* infestation by dwarf mistletoe.

### Sample principal component analysis and correlation assessment

To further investigate the changes of metabolites in plant tissues of *J. tibetica* infested with *A. oxycedri*, broad-targeted metabolomics analyses were performed using LC-MS. The sample correlation heatmap and principal component analysis results indicate that the samples exhibit good biological reproducibility (see Supplementary Appendix A for details), and both of them could clearly differentiate between samples of different groups. The significant changes in the major metabolites in *J. tibetica* as a result of infestation by *A. oxycedri* indicated that *A. oxycedri* had a significant effect on the metabolic regulation of *J. tibetica*.

### Analysis of differential metabolic pathways and differential metabolites

**Figure 6:**
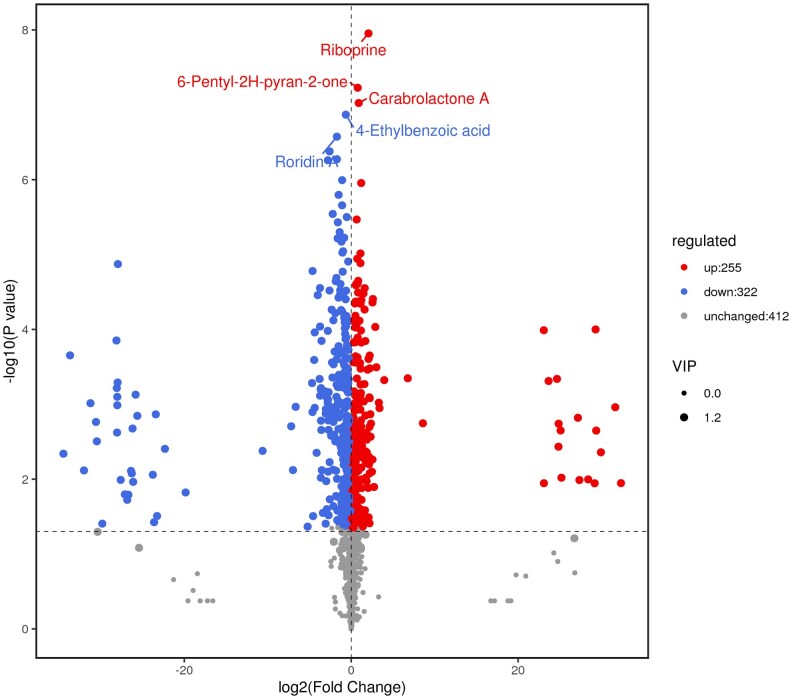
Volcanic map of differential metabolites.

Based on extensive targeted metabolomics analysis, a screening approach combining VIP ≥ 1, fold change (FC) ≥ 1, and univariate statistical analysis [*P* value < .05, false discovery rate (FDR)-corrected], a total of 577 differential accumulated metabolites (DAMs) were identified from 989 metabolites, of which 255 DAMs were up-regulated and 322 DAMs were down-regulated (Fig. 6).

**Figure 7:**
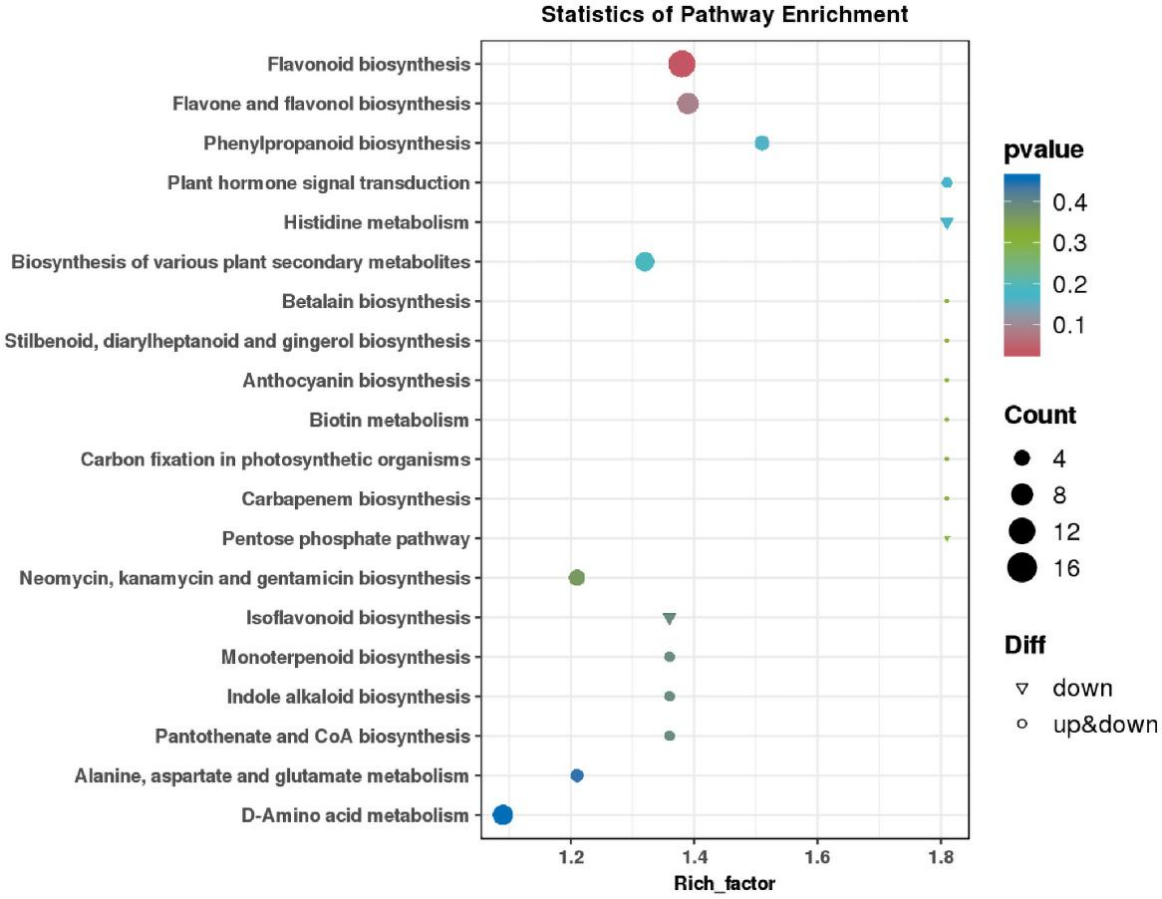
Enriched KEGG pathways of differential metabolites.

KEGG enrichment analyses of DAMs were performed, and pathway enrichment showed that the differential metabolites were mainly enriched in the metabolic pathways of flavonoid biosynthesis (flavonoid biosynthesis), flavone and flavonol biosynthesis and phenylpropanoid biosynthesis (phenylpropanoid biosynthesis) on metabolic pathways (Fig. 7). Differential metabolites such as dihydromyricetin, naringenin chalcone, rhizopodophyllin, chanterellin, prunetin, quercetin, Douglas firin, Pinus pinnatifida, quercetin-1 and rhizopodophyllin were up-regulated in flavonoid biosynthesis pathway, and differential metabolites such as apigenin and 7,4'-dihydroxyflavonoids were down-regulated in the expression pathway (Table 2).

**Table 2:** Differential metabolites of flavonoid biosynthesis.

Code	Compound	Fold change	*P* value	VIP	Type
NEG_q125	Dihydromyricetin	0.72	0.00	1.19	Up
NEG_q255	Naringenin Chalcone	0.45	0.03	1.13	
NEG_q277	Phloretin	0.23	0.01	1.18	
NEG_q123	Dihydrokaempferol	0.42	0.02	1.16	
NEG_q248	Myricetin	0.33	0.00	1.19	
POS_q308	Quercetin	0.38	0.04	1.10	
NEG_q329	Taxifolin	0.47	0.00	1.20	
NEG_q287	Pinocembrin	0.12	0.00	1.20	
NEG_q295	Quercetin-1	0.28	0.01	1.17	
NEG_q278	Phlorizin	0.70	0.01	1.15	
NEG_q76	Apigenin	3.76	0.00	1.17	Down
POS_q54	7,4'-Dihydroxyflavone	1.70	0.03	1.05	

### Combined transcriptome and metabolome analysis

**Figure 8:**
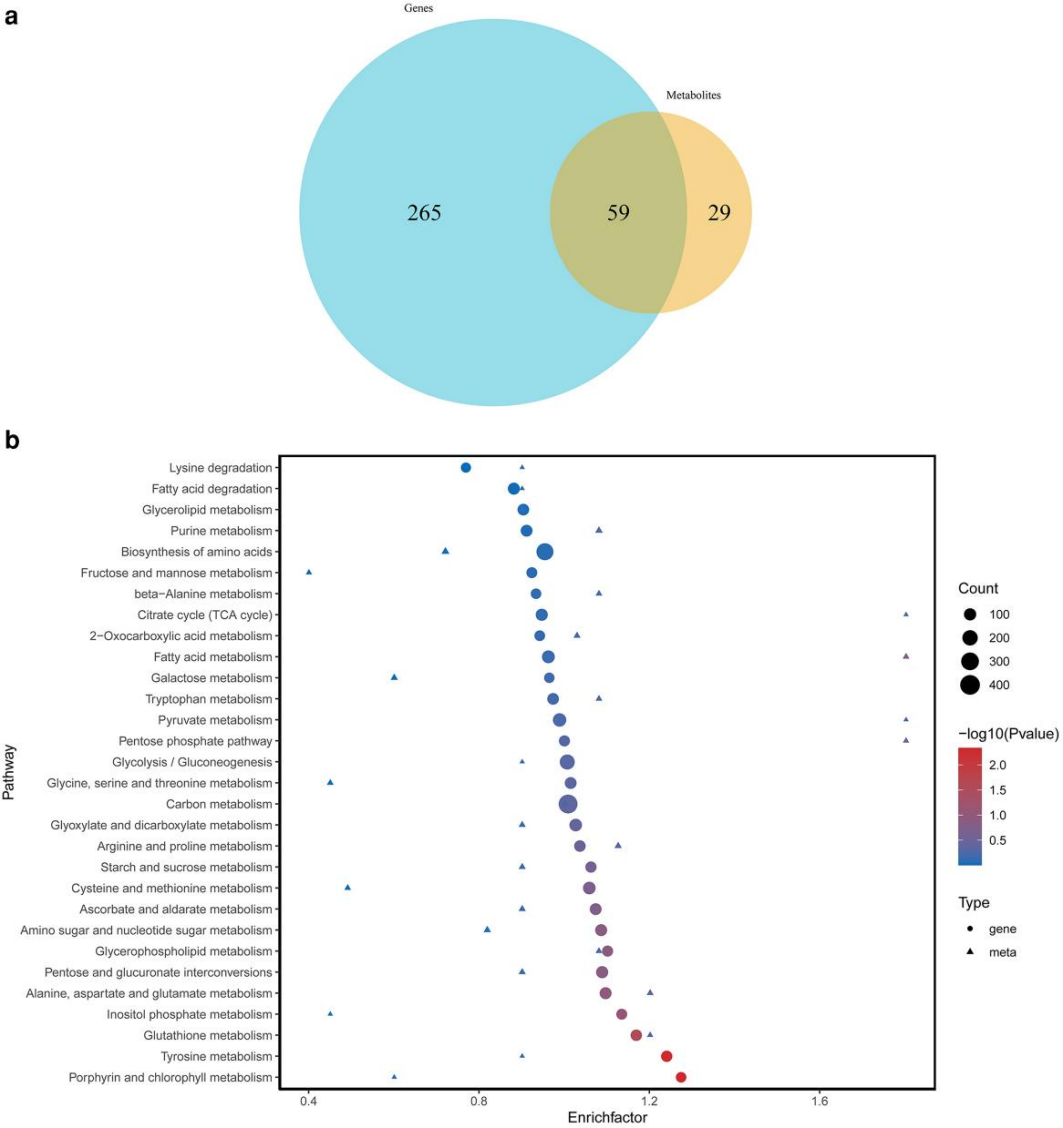
Bubble diagram of enriched pathways shared by transcriptome and metabolome through KEGG analysis. (a) Venn diagram; (b) metabolic pathway analysis. Triangles are DEGs; dots are DAMs. Bigger -lg (*P* value) indicates greater reliable enrichment.

By comparing pathways involving genes in the transcriptome with those involving metabolites in the metabolome, the number of shared pathways was determined. A Venn diagram revealed 59 metabolite pathways showing common enrichment (Fig. 8a). *P*-values were corrected using the Bonferroni method. When the corrected *P*-value (p_bonferroni) ≤ .05, the top 30 co-enriched pathways containing the most differentially expressed genes/metabolites were selected for bubble chart representation (Fig. 8b). This identified porphyrin and chlorophyll metabolism, Tyrosine metabolism, Glutathione metabolism, Inositol phosphate metabolism, Alanine, aspartate and glutamate metabolism, and pentose and glucuronate interconversions. Metabolites and genes within these pathways may be closely associated with the response of *J. tibetica* to *A. oxycedri* infestation.

**Figure 9:**
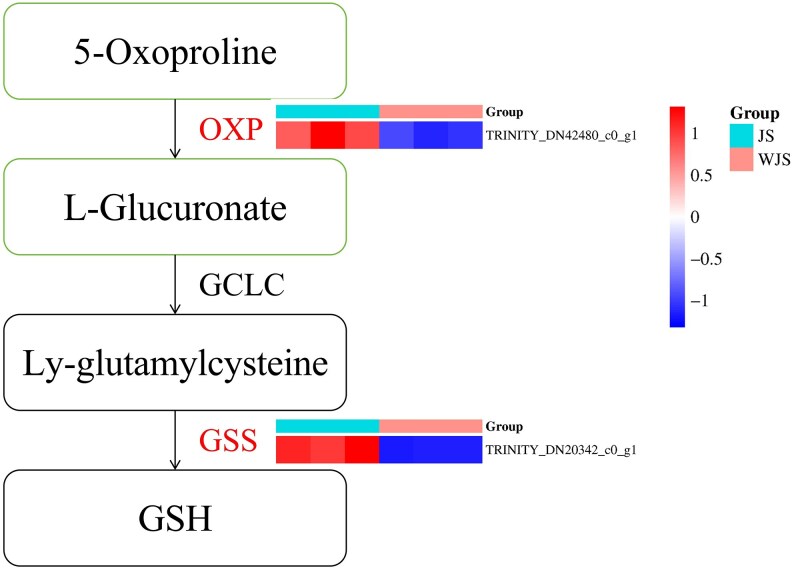
Key DEGs and DAMs in the pathways of glutathione metabolism. Green boxes in the flowchart indicate down-regulated metabolites in JS, Red denotes up-regulated gene expression.

The key DEGs and DAMs in the glutathione metabolic pathway with significant transcriptional-metabolic co-enrichment were mapped (Fig. 9). Expression of genes encoding 5-oxoprolinase (5-oxoprolinase, OXP) and glutathione synthase (GSS) were upregulated in JS samples.5-oxoprolinase (OXP) converts 5-oxoproline to L-glutamate, which is subsequently converted to L-γ-glutamylcysteine, which is then converted to glutathione (GSH) by GSS. The metabolites 5-oxoproline and L-glutamate are reduced.

**Figure 10:**
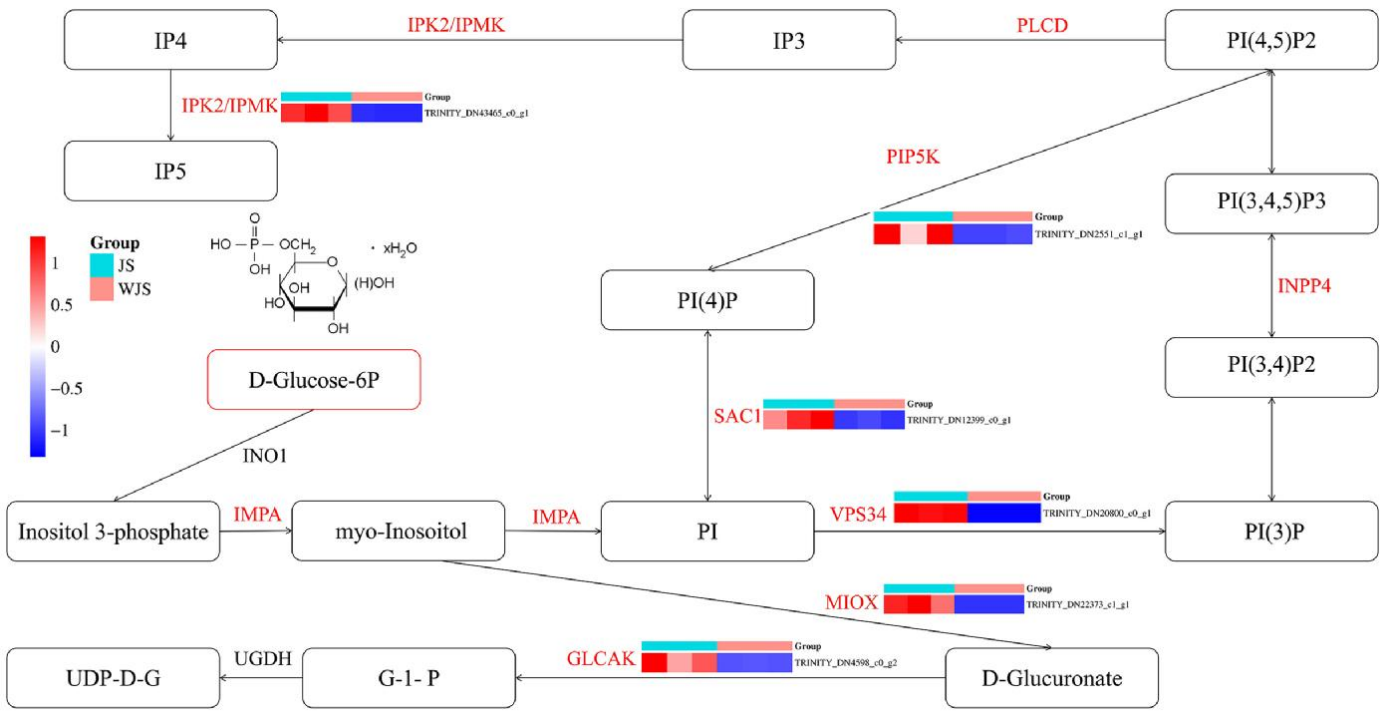
Key DEGs and DAMs in the pathways of inositol phosphate metabolism and pentose and glucuronide interconversion. Red boxes in the flowchart indicate up-regulated metabolites in JS, Red denotes up-regulated gene expression.

Key DEGs and DAMs in the pathways of inositol phosphate metabolism and pentose and glucuronide interconversion, which are significantly co-enriched for transcriptional metabolism, were mapped (Fig. 10). The JS samples encoding inositol-polyphosphate multikinase (IPMK/IPK2), 1-phosphatidylinositol-4-phosphate 5-kinase (PIP5K), phosphatidylinositol 3-kinase (VPS34/PIK3C3), phosphatidylinositol 4-phosphatase (SAC1/SACM1L), inositol oxygenase (MIOX), myo-inositol-1(or 4)-monophosphatase, inositol polyphosphate-4-phosphatase (INPP4), phosphatidylinositol-34,5-trisphosphate 3-phosphatase and dual-specificity protein phosphatase, phosphatidylinositol phospholipase C, delta and glucuronokinase (GLCAK) were upregulated.

Inositol-polyphosphate multikinase (IPK2) phosphorylates inositol 14,5-trisphosphate (1-D-myo-inositol-1,4,5P3,IP3) to inositol 1,4,5,6-tetrakisphosphate (1-D-mo-Inositol-1,4,5P3,IP3). inositol- 1,4,5,6,IP4) and subsequently phosphorylated to 1,3,4,5,6-pentakisphosphatidylinositol (1-D-myo-inositol-1,3,4,5,6,IP5). PI-4-phosphate (PI4P) can be hydrolyzed to phosphatidylinositol by phosphatidylinositol phosphatase SAC1, phosphatidylinositol 5-kinase PI4P can be hydrolyzed to phosphatidylinositol by phosphatidylinositol phosphatase SAC1, and phosphatidylinositol phosphate 5-kinase (PIP5K) converts PI4P to phosphatidylinositol 4,5 diphosphate (PI(4,5)P2), which is a key enzyme in the PI signal transduction pathway.

Increased levels of the metabolite D-glucose 6-phosphate in JS samples shifted the flow of the inositol phosphate metabolic pathway to the pentose and glucuronate interconversion pathway, where glucuronate kinase (GlcAK) converts glucuronate to glucuronate-1-phosphate (D-glucuronate-1P), which is then converted to glucuronate via the inositol oxygenase (MIOX) pathway to UDP -glucuronic acid (UDP-GlcA). The above analyses indicated that glutathione metabolism and inositol phosphate metabolism pathway might be the main secondary metabolic pathways of *J. tibetica* in response to *A. oxycedri* infestation.

### Quantitative Real-Time Reverse Transcription Polymerase Chain Reaction (qRT-PCR) validation of gene expression

**Figure 11:**
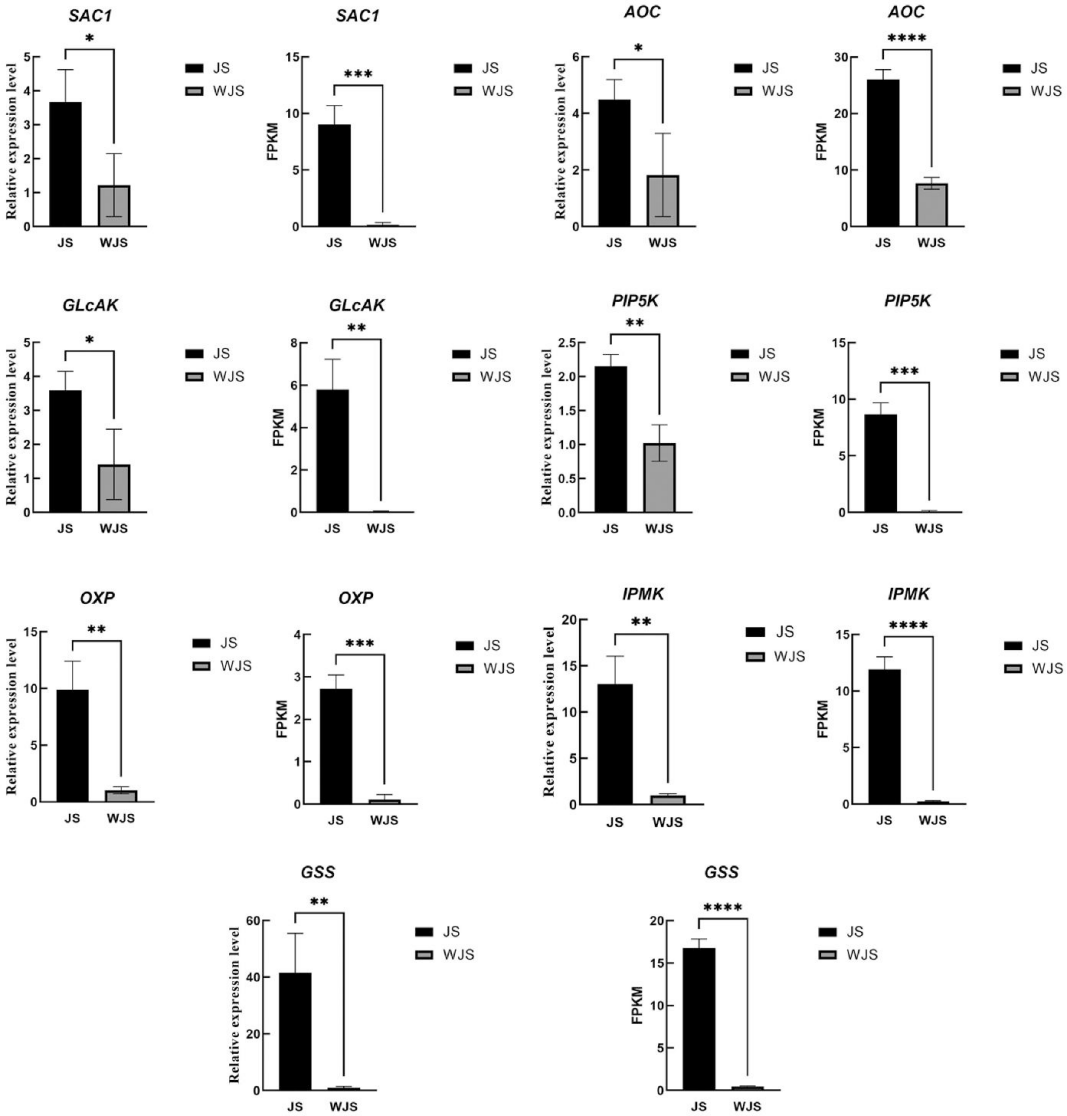
qRT-PCR verification on partial DEGs. *, **, ***, and **** represent JS compared with WJS, respectively The significance levels are *P* < .05, *P* < .01, *P* < .001 and *P* < .0001, respectively.

In order to validate the results of the RNA-Seq assay, seven of the genes from glutathione metabolism were selected for qRT-PCR analysis. They were phosphatidylinositol 4-phosphatase (SAC1,TRINITY_DN12399_c0_g1),1-phosphatidylinositol-4-phosphate-5-kinase (PIP5K,TRINITY_DN2551_c1_g1), inositol-polyphosphate multikinase (IPK2, TRINITY_DN43465_c0_g1), glucuronokinase (GLcAK, TRINITY_DN4598_c0_g2), 5-oxoprolinase (OXP, TRINITY_DN42480_c0_g1), GSS (TRINITY_DN20342_c0_g1), and propadiene oxide cyclase (AOC,TRINITY_DN909_c1_g1). qRT-PCR validation showed that the differential expression trends of these genes were consistent with the results of the RNA Seq assay (Fig. 11), indicating reliable transcriptome data.

## Materials and methods

### Plant material

The test materials were healthy branches and trunks of *J. tibetica* (WJS) and branches and trunks infected with dwarf mistletoe (JS), JS and WJS were collected from the same species in the same region in autumn of 2023 from Nangqian County, Yushu Tibetan Autonomous Prefecture, Qinghai Province, at an altitude of 4230 m. The infested and non-infested branches and trunks were selected on ice and loaded into centrifuge tubes. The infested and non-infested branches were selected on ice and loaded into centrifuge tubes. The collected samples were disinfected with 75% alcohol and treated with sterile water, frozen in liquid nitrogen, and transferred to an ultra-low-temperature refrigerator at −80°C for use. Each group of samples consisted of three replicates.

### Identification of dwarf mistletoe

Symptoms of dwarf mistletoe were observed and morphological features such as shape, size and colour of dwarf mistletoe at different stages of growth were recorded using a stereomicroscope. Genomic DNA was extracted by CTAB method with reference to the literature (Carey et al. 2023). PCR amplification was performed with primer pairs for ITS (1-F3/1-R3), YCf2 and matk genes. The amplified products were sent to Bio for sequencing and BLAST search in GenBank for homology comparison.

### Sample treatment

Six Juniper with uniform growth were selected, of which three were infected by dwarf mistletoe in the experimental group (JS) and three were healthy branches and trunks in the control group (WJS). The samples were washed with distilled water, mixed, and divided into two parts. In the experimental group, the infested and enlarged parts of the branches were intercepted, wrapped in tinfoil and frozen in liquid nitrogen, and sent to Beijing Baimark Biotechnology. The remaining samples were snap-frozen in liquid nitrogen and stored in a refrigerator at −80°C for the determination of physiological indexes, with three biological replicates for each index.

### Measurement of physiological parameters

Physiological parameters were determined using spectrophotometry, including peroxidase (POD), catalase (CAT), polyphenol oxidase (PPO), phenylalanine ammonia-lyase (PAL), cinnamaldehyde dehydrogenase (CAD), and glutathione peroxidase (GSH-Px) activity, as well as malondialdehyde (MDA) and proline (PRO) content, to investigate the physiological response mechanisms between dwarf mistletoe and juniper.

### Transcriptome analysis

A total of six samples from three replicates each of infected and healthy branches were taken for transcriptome analysis, and total leaf RNA was extracted, purified and constructed into sequencing libraries. The raw data (raw data) were filtered using trimmomatic (Wu 2017) software to remove low quality, *N* percentage greater than 10%, and reads with joints, and then filtered and quality controlled by fastqc software to obtain valid data (clean data). Trinity 2.14.0 software was used to assemble and splice the clean data to obtain transcripts, and the longest transcript (Unigene) was selected as the functional gene. Then the software Bowite2 was used to compare the clean data to the Unigene, and RSEM was used for gene expression quantification to obtain the expression values of the genes in different samples, and FRKM was used as the expression quantification index (Xie et al. 2017). The Unigene obtained by splicing was compared with GO and KEGG databases to obtain the annotation information of Unigene. The obtained genes were screened for differentially expressed genes using DESeq2, and genes with FDR (false discovery rate) < 0.01 and FC (fold change) ≥ 2 were designated as differentially expressed, and the differentially significant genes were screened by GO (Gene Ontology) database and KEGG (Kyoto Functional annotation of the genes was performed by Gene Ontology (GO) database and KEGG (Kyoto Encyclopedia of Genes and Genomes) database to analyse the metabolic pathways involved in the differential genes.

### Metabolomic analysis

A total of 6 samples from 3 replicates of infected and healthy branches were taken for broad target metabolome analysis. The broad-target metabolome analyses were performed by Beijing Bemac Biotechnology. The metabolome analysis was performed on a Waters UPLC Acquity I-Class PLUS ultra-high performance liquid chromatograph coupled with an ABSciex Qtrap 6 500 + high-resolution mass spectrometer, and the chromatographic columns used were purchased from Waters Acquity UPLC HSS T3 columns (1.8 μm, 2.1 mm × 100 mm). The raw data obtained were subsequently imported into the R statistical environment for principal component analysis and hierarchical clustering analysis to evaluate the stability and accuracy of the classification model. Differential metabolites were screened using an orthogonal partial least squares discriminant analysis (OPLS-DA) model, selecting those with variable importance in projection (VIP) ≥ 1 and FC ≥ 1, and univariate statistical analysis results (*P* value <.05, FDR-corrected). Model stability was validated through 200 permutations. Differentially expressed metabolites were further annotated and subjected to pathway enrichment analysis using the KEGG database (https://www.kegg.jp/kegg/compound/).

### Real-time fluorescence quantitative PCR validation

Based on the results of transcriptome analysis, seven differentially expressed genes were selected and subjected to real-time fluorescence quantitative PCR (qRT-PCR), and the actin gene (ACT7) was used as an internal reference gene to verify the reliability of its transcriptome sequencing results. The sampling method was consistent with the above, total RNA was extracted using the Trizol method, cDNA was synthesized by reverse transcription, qRT-PCR primers were designed using the PrimerPremier5.0 software (Table 3), and qRT-PCR experiments were carried out, with three replicates for each experiment, and the relative gene expression was calculated using the 2^−ΔΔCt^ method. Histograms of relative gene expression were produced using GraphPad Prism 8.0.2 software. The gene expression of target genes in qRT-PCR and RNA-seq was analysed to verify the reliability of the transcriptome data.

**Table 3:** Genes and primers for qRT-PCR.

Gene	Forward primer (5′–3′)	Reverse primer (5′–3′)
TRINITY_DN12399_c0_g1	ATTGAGAATGTAATGGCCAC	TGGAATCAAACAGGCAAGTC
TRINITY_DN909_c1_g1	CGATTCTTGCTTGTCCACAC	CTATTATGTGCAGGTGTGGG
TRINITY_DN4598_c0_g2	AACAAGATCGAGCTTCATGG	TCCAGGAAACAGTTAAAAGC
TRINITY_DN2551_c1_g1	CTTGATAGCAAGCAACAACC	ATACTTCCCTAACCCCTCAG
TRINITY_DN42480_c0_g1	CAAACACCCCTGATTCAAAG	TGTATCCGTCTCTCCCTCAC
TRINITY_DN43465_c0_g1	TTGAAAGAAAATGTGGTGGG	GATCGAAATGTGGATCACAG
TRINITY_DN20342_c0_g1	ATGCCCCATTTTCCTTATTG	AGTGCTGAAGGAATTCTCCG
ACT7	GGAGGTTCCACCATGTTTCC	GTGCTGAGCGAAGCGAGAAT

## Discussion

When plants are subjected to adversity stress, they produce a variety of stress responses, which are eventually expressed as stress resistance and stress tolerance, and these adaptations and responses can improve the adaptability of plants under adversity. As the most common growth adversity in plants, drought is an important cause of growth inhibition (Lobell et al. 2014), causing damage to leaf structure (Mukarram et al. 2021), and ultimately leading to a decrease in plant biomass and yield (Jia et al. 2020). Dwarf mistletoe, as a perennial semi-parasitic vascular plant, needs to draw water and essential nutrients from the host for its growth and reproduction, disrupting the balance of nutrient and water translocation of the host and causing continuous adverse effects on the health of the host, which ultimately leads to crown dieback and host death (Jiang 2021). It has been shown that dwarf mistletoe parasitism significantly reduces spruce new shoot length, needle length, and leaf centile weight, and the number of new lateral shoots of spruce significantly increases, and spruce new shoots require a large amount of water for growth, and spruce infested with spruce dwarf mistletoe can suffer from water deficiency, which directly leads to an increase in internal MDA content (Lu 2020).

Glutamate, as a multifunctional amino acid, is a precursor of several protein amino acids and influences certain important physiological processes (Galili et al. 2001). It has been shown that glutamate plays an important role in plant response to biotic and abiotic stresses (Forde et al. 2007, Yang 2017). Glutamate administration in drought-stressed kale-type oilseed rape triggers calcium signalling (mainly calcium-dependent protein kinase), which increases salicylic acid biosynthesis and enhances drought-induced PRO accumulation, which in turn regulates cellular redox potential to improve drought tolerance in oilseed rape (La et al. 2020). L-glutamate, as a key structure of glutamate, is biologically important in the metabolism of living organisms, and has more widespread applications. L-glutamic acid reduces salt damage to lentil seedlings by reducing Na+ accumulation, maintaining ionic homeostasis and increasing the activity of antioxidant enzymes (catalase and ascorbate peroxidase), and reduces the oxidative damage of salt stress, resulting in increased survival of lentil seedlings, suggesting that L-glutamic acid has an ameliorative effect on the growth of lentil seedlings (Fardus et al. 2021). glutamate has a role in regulating antioxidant defence mechanisms through glutathione biosynthesis (O'shea et al. 2017). In this experiment, 5-oxoproline was converted to L-glutamate by 5-oxoproline enzyme (OXP) and subsequently to Ly-glutamylcysteine, which was then converted by GSS to glutathione (GSH), OXP (TRINITY_DN42480_c0_g1) and GSS (TRINITY_DN20342_c0_g1) were up-regulated, and the content of 5-oxoproline and L-glutamate was lower than that of non-infested cypress material. The possible reason for this is that when *A. oxycedri* infests *J. tibetica*, it needs to obtain a lot of water from the host to grow, and the lack of water supply leads to the stress of the host, which increases the synthesis of PRO and other antistress substances used to resist the infestation of *A. oxycedri* and leads to the overconsumption of L-glutamate.

Inositol, as an important secondary metabolite, promotes plant growth and development and enhances plant immunity. The phosphatidylinositol (PI) signal transduction pathway is involved in the regulation of seed germination, growth and development, reproduction, senescence and other processes in plants. Phosphatidylinositol phosphate 5-kinase (PIP5K) converts PI4P to phosphatidylinositol 4,5 diphosphate (PI(4,5)P2) (Pertile et al. 1995). PIP5K enzyme (phosphatidylinositol 4-phosphate 5-kinase) is an important phospholipid kinase in organisms that regulates the PI cycle and accelerates the hydroxyl phosphorylation of the 5-position of the 1-phosphatidyl-1D-inositol ring and the phosphorylation of the PI-4 phosphate and the generation of PI-4,5-bisphosphate, a conversion that belongs to the crucial link in the phosphatidylinositol cycle and plays a central role in regulating a variety of signalling pathways (Ling et al. 2007, Khadka et al. 2019, Hansen et al. 2022).

PI4P in the inositol phosphate metabolic pathway reaches the endoplasmic reticulum and can be hydrolyzed to phosphatidylinositol by phosphatidylinositol phosphatase SAC1. It has been shown that AtSAC1 is expressed in all tissues of elongating organs, but mainly in non-elongating parts of the stem such as vascular tissues, and is required for normal cell morphogenesis, cell wall synthesis, and actin organization. The possible mechanism is that SAC1 (phosphatidylinositol 4-phosphatase) affects the dynamics of intracellular phosphatidylinositol, especially PI(4)P and PI(4,5)P2, by influencing the intracellular phosphatidylinositol distribution, maintains the normal function of the secretory pathway, and ensures that cell wall precursors and related enzymes synthesized on the endoplasmic reticulum are efficiently transported to the cell surface for cell wall synthesis and assembly (Zhong et al. 2005).

In the inositol phosphate metabolic pathway, the inositol polyphosphate kinase IPK2 phosphorylates inositol 14,5-trisphosphate IP3 to IP4 and subsequently to IP5 (Frederick et al. 2005) to synthesize phytate (phytate). It has been reported that IPK2 is associated with plant responses to abiotic adversities such as salt stress, and the transcript levels of various stress-responsive marker genes were increased in *trans*-salt mustard ThIPK2 plants under salt stress (Yang et al. 2008), which demonstrated that ThIPK2 has the ability to enhance plant resistance to drought and low-temperature stress (Zhu et al. 2009). Inositol polyphosphate synthesis begins with glucose 6-phosphate (G-6-P), which is converted to inositol-3-phosphate [Ins(3)P] by inositol phosphate synthase. This process is conserved in all living cells (Torabinejad et al. 2006). In plants, Ins(3)P can be further synthesized into InsP6 via a series of phosphorylations via 2 pathways, the inositol lipid dependent pathway and the inositol lipid independent pathway. In this study, the inositol lipid dependent pathway was used to synthesize InsP6, which was first found in large quantities in plant seeds and is thus commonly known as phytate. It has been shown that InsP6 is directly involved in the regulation of plant resistance to adversity stresses such as drought, salt, and disease and intracellular Ca^2+^ content (Gillaspy 2011).

In this study, PIP5K (TRINITY_DN12399_c0_g1), SAC1 (TRINITY_DN12399_c0_g1) and IPK2 (TRINITY_DN43465_c0_g1) genes in the inositol phosphate pathway were significantly up-regulated, and the synthesis of phytate was triggered by the depletion of glucose-6-phosphate, so we hypothesized that up-regulation of phosphatidylinositol 4-phosphatase in the infested cypress may be involved in regulating plant cell wall synthesis and causing the synthesis of InsP6 through regulating intracellular Ca^2+^ content. Phosphatase was up-regulated to stimulate the expression of plant cell wall synthesis genes, and the synthesis of InsP6 may be involved in the regulation of plant resistance to drought and other adversity stresses, and play a messaging role by regulating the intracellular Ca^2+^ content, which caused an instantaneous increase in cytoplasmic Ca^2+^ concentration.

Increased levels of the metabolite D-glucose 6-phosphate shifted the flow of the inositol phosphate metabolic pathway to the pentose and glucuronide interconversion pathway. UDP-D-GlcA is an important precursor molecule for non-cellulosic polysaccharides of the plant cell wall, and approximately 50% of the cell wall material is derived from the precursor UDP-D-GlcA (Zablackis et al. 1995). As an important enzyme in the cell wall precursor UDP-GlcA synthesis pathway, its involvement in the regulation of cell wall development has been reported in Arabidopsis thaliana (Pieslinger et al. 2010), and AtGlcAK mutants exhibit hypersensitivity to ABA and reduced root development under water stress, making the plant more susceptible to drought stress (Xiao et al. 2017). GlcAK (TRINITY_DN4598_c0_g2) was significantly up-regulated in the differential gene expression of *J. tibetica*, presumably *J. tibetica* was parasitically infested by *A. oxycedri* to resist the stress through the enhancement of cell wall synthesis pathway. From the above analysis, it is evident that the primary enrichment pathways activated in *J. tibetica* in response to *A. oxycedri* are all associated with defence against drought stress. It is hypothesized that the growth and reproduction of the *A. oxycedri* necessitate the extraction of substantial quantities of water and essential nutrients from the host. This parasitism disrupts the host's transport balance of nutrients and water, and further leads to the proliferation of numerous adventitious branches on the *J. tibetica* plant. The development of new lateral branches demands substantial water resources, thereby exacerbating water deficiency in the infected *J. tibetica* plants.

Following infection by *A. oxycedri*, differentially expressed metabolites in Juniper are primarily concentrated in metabolic pathways such as flavonoid biosynthesis, flavone and flavonol biosynthesis, and phenylpropanoid biosynthesis. Flavanols, as a class of secondary metabolites widely present in plants, play a key role in regulating plant growth and development, flower colour formation, and stress responses (Zhang et al. 2025). Dihydromyricetin (DMY) belongs to the flavonoid class, PAL, as the key initiator enzyme in DMY biosynthesis, catalyses the conversion of phenylalanine to cinnamic acid, 4-cinnamoyl-CoA ligase (4CL), and 4-cinnamoyl-CoA monooxygenase (CYP73A), which catalyse the conversion of cinnamic acid to *p*-cinnamoyl-CoA. Chalcone synthase (CHS) and chalcone isomerase (CHI) are key enzymes in the upstream of the second stage, catalysing the formation of naringin from *p*-cinnamoyl-CoA. Naringin is the first stable intermediate in the flavonoid pathway (Li et al. 2024), from which dihydroquercetin is ultimately generated via a series of catalytic enzymes, including F3H (3 pathways), F3′H (1 pathway) and F3′5′H (3 pathways) (Gu et al. 2022). Previous studies have shown that in the hairy roots of *Astragalus membranaceus*, the transcription factor AmMYB35 can specifically bind to the promoter region of AmFLS, thereby activating its transcriptional expression and promoting the biosynthesis of flavonoid compounds (Qi et al. 2025). As important antioxidants, flavanols can alleviate oxidative damage by scavenging excess reactive oxygen species accumulated under abiotic stress, thereby effectively enhancing the plant’s drought tolerance.

In this study, the two types of flavonoids identified as differentially expressed dihydromyricetin and naringin chalcone were both significantly up-regulated. As key products in the flavonoid biosynthetic pathway, they enhance the resistance of *Juniperus chinensis* to *Juniperus parasitica* infection by scavenging the excessive ROS accumulated during stress. The flavonoid biosynthesis pathway is closely associated with the response of Juniper to *A. oxycedri*.

The process of plant-pathogen interactions causes adversity to the plant, inducing a specific response that leads to changes in defence enzymes such as POD, CAT, PPO, PAL and CAD in the plant. Sharp increase in reactive oxygen species (ROS) is a common manifestation in plants facing various adversity stresses, indicating that ROS play a very important role in plant adversity response (Peng et al. 2002, Qi et al. 2017). POD, CAT, PPO, etc., are associated with scavenging ROS (Li et al. 2012). It has been shown that parasitizing sunflower by *Orobanche cumana* produces large amounts of ROS, leading to membrane lipid peroxidation and cell membrane damage (Li et al. 2019). ROS scavenging mechanisms were activated in all sunflower susceptible and resistant materials parasitized but with different roles. Antioxidant enzymes were accumulated in sunflower and their activities were significantly increased in comparison to the control after inoculation with *Orobanche cumana* (Yang et al. 2017). Glutathione reductase is a flavoprotein oxidoreductase that catalyses the reduction of oxidized glutathione (GSSG) to reduced glutathione (GSH). A higher GSH/GSSG ratio enhances the clearance of ROS generated during oxidative stress, making GSH-Px enzyme activity a crucial indicator of oxidative stress resistance. The increase in GSH-Px enzyme activity under stress conditions enhances plant tolerance (Foyer et al. 1997, Chen et al. 2015). The findings of this study are consistent with those.

The products of phenylalanine deaminase (PAL) are direct or indirect precursors of a wide range of secondary metabolites, and the secondary products produced are of great importance in many aspects of plant response to stress, antistress defences, and improved antioxidant capacity (You et al. 2020). Cinnamyl alcohol dehydrogenase (CAD) has a key role in lignin synthesis. The results of physiological indexes showed that the physiological indexes of the infested branches were higher than those of the corresponding non-infested cypress branches to different degrees, which was consistent with the results of other studies. Brahmi et al. observed that resistant chickpea mutants exhibited elevated levels of soluble phenolic compounds, phenylalanyl aminopeptidase (PAL) activity, guaiacol peroxidase activity, and PPO activity when studying the parasitism of *Orobanche foetida* Poir. on radiation-induced chickpea mutants (Brahmi et al. 2016). Extensive research indicates that increased PAL activity leads to heightened phytoalexin synthesis, elevated lignin content, and increased phenolic compound levels (Friend et al. 1973, De Jaegher et al. 1985, Sewalt et al. 1997). The findings of this study are consistent with those.

Malondialdehyde (MDA) is produced when biological tissues or organ membranes undergo lipid peroxidation in response to adverse conditions. Its concentration correlates closely with the extent of stress-induced damage to the organism. When plants grow in arid environments, free radicals or ROS accumulate extensively within the plant body. Upon reaching a certain threshold, this triggers lipid peroxidation, producing the harmful substance MDA. This leads to intracellular electrolyte leakage, elevated relative electrical conductivity, ultimately disrupting the structure and function of the cell membrane. (Kang et al. 2024). The findings of this study are consistent with those.

The above results indicate that various physiological indicators of *J. tibetica* branches and stems exhibit positive responses under *A. oxycedri* parasitism. Although unable to resist dwarf mistletoe infestation, the plant counteracts its impact by enhancing the activity of antioxidant-related defence enzymes, increasing lignin content, and improving drought-related physiological indicators.

## Conclusion

In this study, we used the cypress branches of parasitized by *A. oxycedri* (JS) and not parasitized by *A. oxycedri* (WJS) as experimental materials to investigate the physiological and molecular response mechanisms of infested cypress, and we mainly obtained the following conclusions.

This study found that infection by *A. oxycedri* significantly increased the activity of five physiological indicators (POD, CAT, PPO, PAL, CAD, GSH-Px MDA, and PRO) in the branches and stems of large-fruited juniper. Although infected plants cannot resist dodder infestation, they effectively counteract parasitic effects by enhancing antioxidant defence enzyme activity, increasing lignin content, and improving drought-tolerance physiological indicators.

Through morphological observation and molecular biological identification, the species of dwarf mistletoe parasitizing the branches and stems of *J. tibetica* was preliminarily determined to be *A. oxycedri*. Transcriptome and metabolome analyses revealed significantly co-enriched pathways including porphyrin and chlorophyll metabolism, tyrosine metabolism, glutathione metabolism, inositol phosphate metabolism, alanine, aspartate, and glutamate metabolism, and pentose-glucuronic acid interconversion. Eleven DEGs and three DAMs associated with glutathione and inositol phosphate metabolism pathways were screened. Among these, DEGs including OXP1, GSS, IPMK/IPK2, PIP5K, SAC1, and GLCAK were upregulated in infected juniper, One DAM, D-glucose-6-phosphate, accumulated in infected junipers, while DAMs such as L-glutamic acid and L-pyroglutamic acid decreased in infected Thuja orientalis. This indicates that the primary enrichment pathways activated in *J. tibetica* in response to *J. tibetica* parasitic infection are associated with drought stress resistance. It is speculated that this may occur because *J. tibetica* mistletoe absorbs large amounts of water from the host *J. tibetica*, leading to water deficiency in the host and triggering drought stress-related response mechanisms.

## Supplementary Material

plag022_Supplementary_Data

## Data Availability

The raw data are available as supporting information (Appendix: All gene expression).
