# Supplementary material for: Joint transcriptomic and metabolomic analyses of the mechanisms of Juniperus tibetica in response to Arceuthobium oxycedri
Source: AoB Plants. 2026 May 21;18(3):plag022. doi: 10.1093/aobpla/plag022 (PMC13267142; doi:10.1093/aobpla/plag022)
Supplement: plag022_Supplementary_Data [file plag022_supplementary_data.zip › New Microsoft Word Document.docx]

**Appendix A** Heatmap of Pearson correlation between samples and principal component analysis on samples


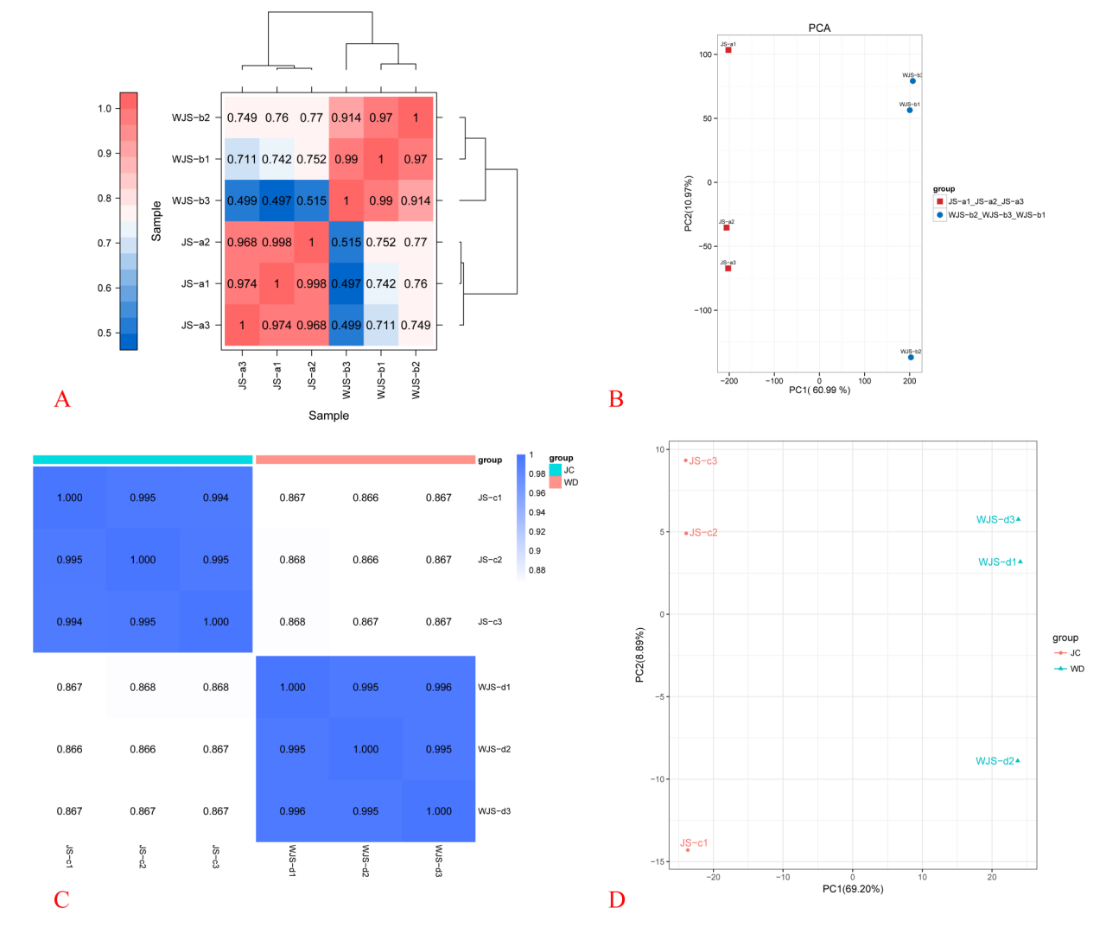


A: heatmap of Pearson correlation between transcriptome samples; B: principal component analysis on

transcriptome samples; C: heatmap of Pearson correlation between metabolome samples; D: principal component analysis on metabolome samples.
