# Supplementary figures and images for: Joint transcriptomic and metabolomic analyses of the mechanisms of Juniperus tibetica in response to Arceuthobium oxycedri
Source: AoB Plants. 2026 May 21;18(3):plag022. doi: 10.1093/aobpla/plag022 (PMC13267142; doi:10.1093/aobpla/plag022)

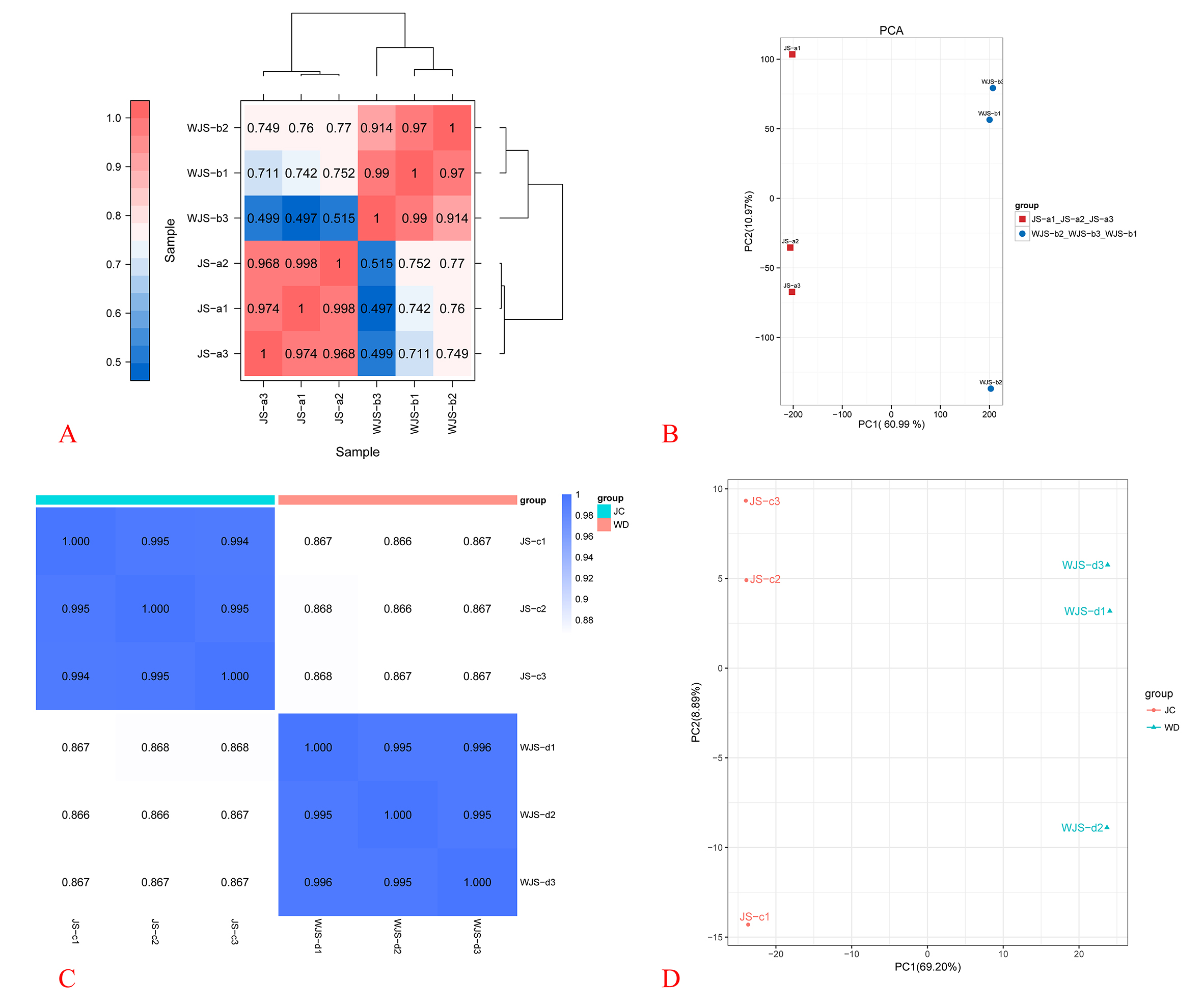

Supplement: plag022_Supplementary_Data [file plag022_supplementary_data.zip › Appendix A.jpg]
